# Supplementary material for: Efficacy of federated learning on genomic data: a study on the UK Biobank and the 1000 Genomes Project
Source: Front Big Data. 2024 Feb 29;7:1266031. doi: 10.3389/fdata.2024.1266031 (PMC10937521; doi:10.3389/fdata.2024.1266031)
Supplement: Supplementary file 1 [file Data_Sheet_1.PDF]

Efficacy of federated learning on genomic data: a study on the UK Biobank and the 1000 Genomes Project

Dmitry Kolobkov[1, 2, *, †], Satyarth Mishra Sharma[1, 3, *], Aleksandr Medvedev[1, 3], Mikhail Lebedev[1], Egor Kosaretskiy[1], and Ruslan Vakhitov[1]

[1]GENXT, Hinxton, UK
[2]Vavilov Institute of General Genetics
[3]Skolkovo Institute of Science and Technology
[†]Corresponding author, dmitry.s.kolobkov@gmail.com
[*]Equal contribution

Supplementary materials

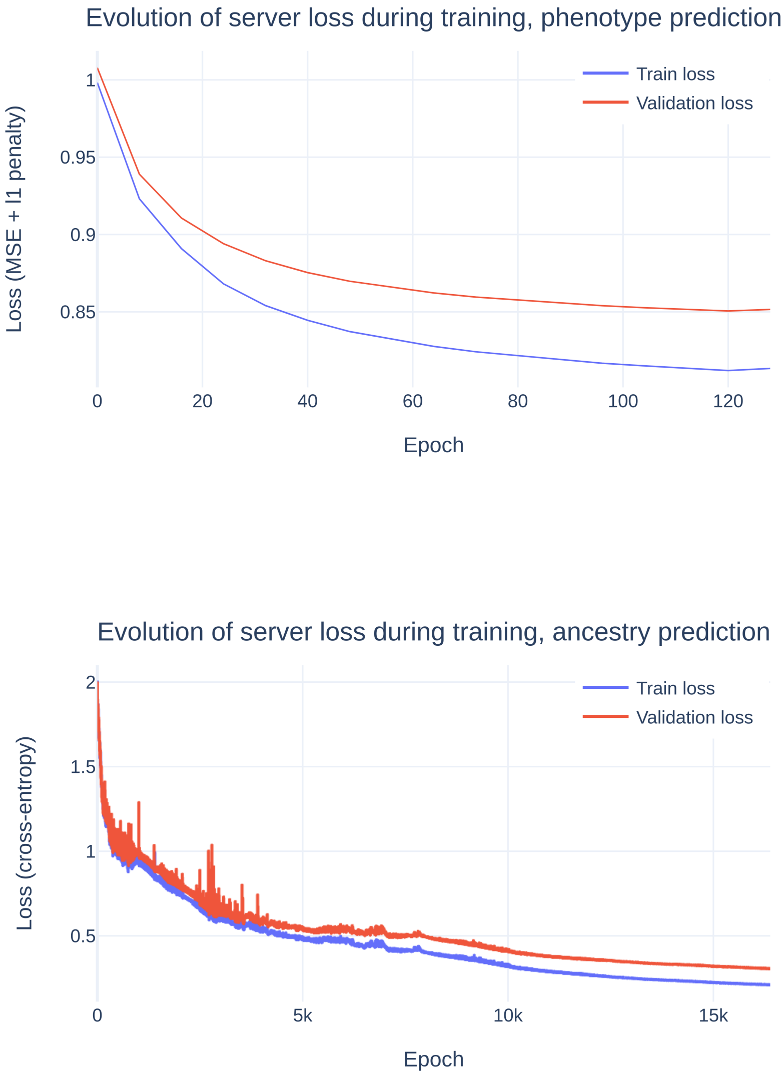

Figure S1: Representative loss curves showing the evolution of training and validation loss during the training process.

Node	Sample count	Variants after QC	Features
Barts	12067	350832	10000 top GWAS SNPs + age + sex
Birmingham	22396	348321	10000 top GWAS SNPs + age + sex
Bristol	42068	347333	10000 top GWAS SNPs + age + sex
Bury	20557	347936	10000 top GWAS SNPs + age + sex
Cardiff	17596	347038	10000 top GWAS SNPs + age + sex
Cheadle	12905	347510	10000 top GWAS SNPs + age + sex
Croydon	25934	349756	10000 top GWAS SNPs + age + sex
Edinburgh	15555	346833	10000 top GWAS SNPs + age + sex
Glasgow	17613	347407	10000 top GWAS SNPs + age + sex
Hounslow	26997	346990	10000 top GWAS SNPs + age + sex
Leeds	37169	347516	10000 top GWAS SNPs + age + sex
Liverpool	26354	347717	10000 top GWAS SNPs + age + sex
Middlesborough	18816	347540	10000 top GWAS SNPs + age + sex
Newcastle	32285	347067	10000 top GWAS SNPs + age + sex
Nottingham	30125	347918	10000 top GWAS SNPs + age + sex
Oxford	13585	347208	10000 top GWAS SNPs + age + sex
Reading	28361	346857	10000 top GWAS SNPs + age + sex
Sheffield	24353	347821	10000 top GWAS SNPs + age + sex
Stoke	16466	347374	10000 top GWAS SNPs + age + sex

Table S1: Statistics for the UK Biobank dataset split into nodes according to the assessment centre of the participants.

Node	Sample count	Variants after QC	Features
AFR	682	871307	20 PCs
AMR	365	871307	20 PCs
EAS	509	871307	20 PCs
EUR	540	871307	20 PCs
SAS	528	871307	20 PCs

Table S2: Statistics for the 1000 Genomes dataset split into nodes by participant superpopulation.
